# Recent Work upon Paroxysmal Hæmoglobinuria

**Published:** 1906-01-27

**Authors:** 


### RECENT WORK UPON PAROXYSMAL HÆMOGLOBINURIA.

Paroxysmal hæmoglobinuria is a rare and mysterious disorder. It is characterised, as is well known, by the occasional passage of bloody urine in which the colouring matter of the blood appears dissociated from its normal vehicles, the red corpuscles. The disease is more frequent in males than females and occurs chiefly in adults, according to Osler. It is especially associated with cold and exertion, and may be readily induced by these influences in the case of a susceptible person. There appears to be an association, at present not understood, between this variety of hæmoglobinuria and Raynaud's disease. Osler, however, thinks the connection a rare one. The attacks come on suddenly and may or may not be attended with such evidences of constitutional disturbance as chills or fever, vomiting or diarrhœa. The attacks are short-lived, so short-lived as to allow the occurrence of two distinct seizures in one day, with an interval of bloodless urination.

The essential pathology of this strange disorder is not known, and many hypotheses have been lavished upon the subject. On the whole the balance of opinion has favoured the view that the hæmoglobinuric paroxysm is an indication of an acute toxæmia acting upon patients affected with a chronic alteration of the blood. The most recent work upon the matter has been carried out by Dr. J. Eason.[1] This author has approached the problem of the causation of the disease on an assumption suggested by recent advances in the study of immunity. This assumption is to the effect that the hæmolytic poison assumed to be at work consists of two elements, the toxic element itself, or complement, and an intermediary body (the amboceptor of Ehrlich) by the assistance of which the toxic element is enabled to enter into chemical combination with the red blood cell.

Dr. Eason's investigations were carried out upon two patients, one of whom was a particularly appropriate subject for the purpose, since a paroxysm could be induced with almost unfailing certainty merely by allowing him to be up and about for a couple of hours. The technique was briefly as follows. By the use of cantharides a blister was raised measuring about two and a half inches square, and containing approximately 10 c.c. of serum. This was, under strict aseptic precaution, punctured and drained into a sterile test-tube. In order to test the toxicity of this serum to the red-blood cells of the same individual or another, one cubic centimetre was placed in a test-tube and to it were added three drops of the blood under consideration, obtained direct and aseptically after a puncture of the finger. The tube was then gently shaken and allowed to stand for several hours.

The first experiment was as follows: The patient was allowed to be out of bed for two hours. At the end of this interval he began to shiver, and the characteristic symptoms of a paroxysm immediately ensued. A blister was applied to the abdomen and the serum obtained on the following day. This was, therefore, the serum of an interval. For the purposes of controlling the experiment serum was in a similar manner obtained from a patient convalescent from acute rheumatism, and from a girl suffering from nephritis. The bloods to be tested were derived from three healthy subjects, one of whom was the rheumatic convalescent above mentioned. Portions of the serum of the hæmoglobinuric patient were mixed with three drops of each of the three bloods respectively; in each case the result was a tinting of the serum to a deep carmine colour, while the control sera remained either quite pale or only very slightly tinted. Further, the hæmoglobinuric serum (henceforward to be called Hb. S.) formed a clot far less dense than was the case with the other sera, while the microscopic features of the blood exposed to the Hb. S. for ninety minutes differed from the controls in the absence of rouleaux, in the greater deformity of the red cells, and in the unevenness of their colouring. The small cells were deeply coloured, the larger ones very pale.

The next experiment was so arranged that serum of the paroxysm should be employed. Accordingly a blister was applied while the patient was in bed. Five hours later he was allowed to get up. Four hours later again he began to shiver, and presently passed dark urine containing hæmoglobin. The serum thus obtained, when mixed with normal blood, resulted in hæmolysis, a phenomenon which was still more obvious when the blood examined was that of the patient himself, obtained during an interval. Control experiments with normal sera were negative in each case.

The actual microscopic appearances of blood undergoing this hæmolysis are the following: There is apparent a great variation in the size and colouring of the red cells, as has been stated; in addition there may be seen in numerous cells globules of dissolved hæmoglobin. Occasionally such globules may be seen lying free in the serum, apparently so recently extruded from a cell that sufficient time has not yet elapsed for their solution in the serum. Dr. Eason was also able to corroborate the observation of Levaditi that phagocytic leucocytes will attack with extraordinary avidity, and subsequently ingest, red blood cells which are held to contain the intermediary bodies by the aid of which union is achieved between hæmolytic toxins and the cells to be affected. Ruziczka has found that in the case of a guinea-pig immunised against fowl's blood, the phagocytic leucocytes of the guinea-pig are able to destroy the red corpuscles of the fowl without previously absorbing them; in such a case the leucocytes attach themselves to the red cells, and digest the latter little by little, as though actually gnawing or nibbling them.

A series of experiments was subsequently carried out with a view to determining the effect of temperature upon the process of hæmolysis by the serum of paroxysmal hæmoglobinurics. The result of these experiments was to show that all preparations which were kept at the blood temperature showed no signs of hæmolysis, though the latter phenomenon was well marked after exposure of the preparation for some time to the temperature of the room.

The general conclusions drawn by the author are as follows:—

1. A pathological substance is present in the blood, serum, and lymph of individuals affected with paroxysmal hæmoglobinuria.

2. This substance can dissolve (in vitro) the corpuscles of the affected individual (autolysis), and those of normal individuals (isolysis) provided that the conditions of temperature be suitable.

3. Temperature conditions in relation to hæmolysis require further study: it is, however, apparent that a temperature much below that of the blood favours the action of the hæmolytic substance, while the normal body temperature retards or prevents it.

4. The observations above considered corroborate those of Levaditi, Gruber, Ruziczka and others, regarding the activity of phagocytic leucocytes during hæmolysis in vitro.

5. These authors consider that the excessive phagocytic action observed is significant of an antecedent union of an intermediary body with the red corpuscles.

6. The intermediary body of paroxysmal hæmoglobinuria cannot, however, of itself produce solution of red corpuscles: for this it is necessary that there should also be present a thermo-labile toxic element, the complement. (This latter is destroyed by exposure to a temperature of 56° C. for one hour, while the intermediary body is thermo-stabile and survives such an exposure).

7. The changes observed in the red cells in course of solution correspond with those produced during hæmolysis by an immune serum.

8. The serum of normal individuals does not cause hæmolysis. The serum of various individuals suffering from disease produces either little hæmolysis, or (more often) none at all.

[1] Edin. Med. Jour., Jan., 1906.

